# Current Progress and Future Trends of Genomics-Based Techniques for Food Adulteration Identification

**DOI:** 10.3390/foods14071116

**Published:** 2025-03-24

**Authors:** Jing Zhao, Wei Yang, Hongli Cai, Guangtian Cao, Zhanming Li

**Affiliations:** 1 School of Grain Science and Technology, Jiangsu University of Science and Technology, Zhenjiang 212100, China; 2 Guangdong Provincial Key Laboratory of Aquatic Products Processing and Safety, College of Food Science and Technology, Guangdong Ocean University, Zhanjiang 524088, China; 3 College of Life Sciences and Medicine, Zhejiang Sci-Tech University, Hangzhou 310018, China; 4 College of Standardisation, China Jiliang Universtiy, Hangzhou 310058, China

**Keywords:** food adulteration, high-resolution melt analysis, DNA barcoding, food authentication, CRISPR–Cas system

## Abstract

Addressing the pervasive issue of food adulteration and fraud driven by economic interests has long presented a complex challenge. Such adulteration not only compromises the safety of the food supply chain and destabilizes the market economy but also poses significant risks to public health. Food adulteration encompasses practices such as substitution, process manipulation, mislabeling, the introduction of undeclared ingredients, and the adulteration of genetically modified foods. Given the diverse range of deceptive methods employed, genomics-based identification techniques have increasingly been utilized for detecting food adulteration. Compared to traditional detection methods, technologies such as polymerase chain reaction (PCR), next-generation sequencing (NGS), high-resolution melt (HRM) analysis, DNA barcoding, and the CRISPR–Cas system have demonstrated efficacy in accurately and sensitively detecting even trace amounts of adulterants. This paper provides an overview of genomics-based approaches for identifying food adulteration, summarizes the latest applications in certification procedures, discusses current limitations, and explores potential future trends, thereby offering new insights to enhance the control of food quality and contributing to the development of more robust regulatory frameworks and food safety policies.

## 1. Introduction

Food fraud costs the international food industry an estimated USD 10 to USD 15 billion annually, encompassing expenses related to product recalls, declining market share, and increased testing and regulatory costs aimed at addressing adulteration. In January 2025, Auramine O—an industrial dye classified as a Group 2B carcinogen by the World Health Organization and banned in Chinese food—was detected in Thai durian exports. As a result, China required all imported durians to provide mandatory testing reports and undergo batch-by-batch sampling inspections. In response, Thailand intensified its export inspections and ceased purchases from areas prone to such issues. These deceptive practices not only inflict significant economic damage but also pose potential risks to consumer health [1,2]. In October 2024, a top influencer claimed that the sweet potato vermicelli being promoted was “made purely from sweet potatoes” during a live streaming session. However, after sampling and inspection by a third-party testing institution, cassava components were actually detected in the product. The false labeling on this commodity not only misled consumers but also, due to potential health risks such as allergic reactions, seriously violated the provisions of the Food Safety Law regarding the authenticity of food labels. In order to ensure the accuracy of DNA detection technology, it is necessary to conduct thorough research on the genomic sequences of the target species. This includes constructing a comprehensive reference database to cover the genetic diversity within the species and to avoid cross-reactions with the sequences of closely related species. In addition, the design of specific primers or probes relies on high-quality genomic data. Therefore, obtaining sufficient sequence information is crucial for the detection of food adulteration. Adulterated foods can lead to a range of health issues, including diarrhea, abdominal pain, nausea, vomiting, cancer, anemia, liver damage, and other diseases, thereby seriously threatening public health and contributing to economic losses and social instability [3,4,5].

Food adulteration and fraud increasingly involve various aspects, including meat, aquatic products, milk and dairy products, oils, flour, berries, honey, and other foods. The methods of food adulteration and fraud are becoming more diverse and covert [6,7]. These practices are primarily manifested in the use of cheaper, lower-quality animals or plants to replace those with higher commercial value, the substitution of inferior varieties for high-quality ones, and the misleading labeling of inexpensive foods as premium products or enhancements to their appearance [8,9]. Consequently, effectively and swiftly identifying food ingredients and combating food adulteration and fraud has become a focal point of research.

The demand for complete, accurate, and reliable food information is not only a primary concern for consumers but also an urgent need for industry stakeholders. As a result, the pursuit of fast, accurate, and sensitive food adulteration detection technologies, along with the establishment of a safe food market environment, aims to protect consumer health and uphold the collective interests of the industry and government [10]. Compared with genomics technology, chromatography and mass spectrometry, which have similarities in chemical composition, generally have lower sensitivity in detecting this kind of adulteration [11]. In addition, these methods usually take a long time and require complex extraction procedures, and the equipment is relatively expensive, which hinders the promotion of their practical applications in the food industry [12]. In recent years, the field of food adulteration identification has witnessed the widespread adoption of omics technologies, driven by rapid advancements in molecular biology and related disciplines [13,14]. These techniques provide a direct reflection of biological gene sequence variations, independent of factors such as the geographical environment, processing conditions, and storage time, and have greatly promoted the level of food quality assurance and food safety supervision.

**Figure 1:**
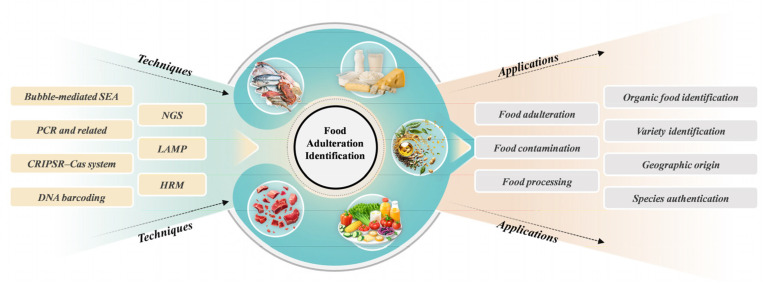
Illustration of genomics approaches for identification of food authenticity and food fraud.

Over the past few years, the emergence of genomics analysis techniques has significantly enhanced the application of genomics technology in combating food adulteration (Figure 1). By designing specific primers and probes to amplify and detect the mitochondrial or genomic DNA of target species, these technologies enable the rapid and accurate identification of food authenticity [15,16]. The application of these adulteration detection methods provides essential technical support for ensuring food safety and is regarded as one of the most effective approaches for verifying food authenticity [17].

We systematically reviewed the research progress of various genomics technologies in the field of food adulteration detection and explored the potential future development directions of these technologies. They have played a crucial role in identifying illegal ingredients adulterated in food, tracing the sources of raw materials, and other related aspects. Based on the current research achievements, we look ahead to the future and propose that genomics technologies may develop towards integration with rapid detection methods, spectroscopic techniques, and deep learning. This would better equip us to address the increasingly complex challenges of food adulteration and ensure food safety.

The ongoing evolution of the modern food industry has resulted in the introduction of numerous new food products with increasingly complex compositions. As a consequence, there is a pressing need for both qualitative and quantitative detection of various components to effectively address the challenges posed by food adulteration and fraud. This review aims to present recent advancements in genomic technologies, summarize their current development, limitations, and application scope, and compile specific applications across different types of food. Technologies such as PCR, NGS, HRM analysis, DNA barcoding, and the CRISPR–Cas system are emphasized for their accuracy and sensitivity in identifying food adulteration and fraud. By providing an overview of the latest research advancements in food certification, this review intends to offer new insights for combating food adulteration and fraud.

## 2. Genomics Approaches

### 2.1. Traditional PCR Technology and Its Extensions

PCR and its extensions based on nucleic acid detection are widely employed in food identification. In recent years, advances in gene detection technology have led to the development of various conventional PCR-based methods, including real-time fluorescence quantitative PCR, multiplex PCR, restriction fragment length polymorphisms (RFLPs), droplet digital PCR (ddPCR), fluorescent probe technologies, and PCR biosensor combinations, which have been applied in adulteration identification, varietal identification, and origin traceability in multiple food products. These methods utilize specific primers designed based on genetic sequence variations among different species, enabling the amplification of targeted DNA bands for the detection, analysis, and determination of food component sources [18,19].

Currently, conventional PCR technology is well established, with advantages including simple operation, short processing time, rapid results, and ease of use. However, standard PCR does not provide accurate quantification, resulting in uncertain outcomes. Its detection limit is usually at the nanogram level, and the sensitivity is relatively low, making it suitable for experiments that require a large amount of template DNA. Cleaved amplification polymorphism sequence-tagged sites (CAPs), also known as PCR–restriction fragment length polymorphism (PCR-RFLP), are based on conventional PCR, where amplified bands are digested by endonucleases to detect their polymorphism.

Conventional PCR can only amplify specific DNA fragments and cannot distinguish species with similar sequences. PCR-RFLP combines PCR amplification with restriction endonuclease digestion, allowing for genotyping based on sequence differences. This method offers good cost-effectiveness but has a relatively low throughput. Moreover, not all single-nucleotide polymorphisms (SNPs) are suitable for detection using this method. The detection limit of PCR-RFLP typically ranges from the picogram to nanogram levels, with moderate sensitivity, making it suitable for detecting specific sequence variations.

qRT-PCR is a highly sensitive method capable of monitoring changes in RNA expression levels in real time. Although it is more expensive than traditional PCR, its high precision and sensitivity make it widely used in gene expression research and clinical diagnosis. The detection limit of qRT-PCR can reach the femtogram level, and its sensitivity is extremely high, making it suitable for analyzing trace samples and detecting rare transcripts. Additionally, it can rapidly and efficiently identify dairy products from different animal sources.

Therefore, it is necessary to select an appropriate PCR method based on different situations. Table 1 summarizes the characteristics, advantages, and disadvantages of traditional PCR and the technologies derived from it.

#### 2.1.1. PCR-RFLP

PCR-RFLP is simple, rapid, and low-cost. It can identify the source of milk through PCR amplification, enzymatic digestion, electrophoresis, and imaging, but it cannot perform quantitative analysis. Multiplex PCR has the advantages of high efficiency, high throughput, and low cost. It can amplify multiple targets in a single reaction to identify multi-source dairy products. For example, it can detect the animal origin in laboratory-made cheese and commercial Italian cheese, with a detection limit of 0.1–0.5%. The disadvantage is that false positive results may occur. Real-time PCR has high sensitivity, strong specificity, and good consistency. It can perform qualitative and quantitative analysis. The SYBR Green-based method is economical and rapid, while the TaqMan-based method has high specificity and can set multiple detection targets. For example, TaqMan probes can be used to detect the DNA of different species in milk, with a detection limit as low as 0.001 ng/μL. The SYBR Green method has low specificity and requires melting curve analysis. Digital PCR has a sensitivity at the single-molecule level, has a detection limit as low as 0.001%, can perform absolute quantification, and is not affected by Ct values, PCR amplification efficiency, and inhibitors. For example, it can be used to detect the milk source in cheese. The disadvantages are a high cost and a narrow dynamic range [24]. Consequently, some low-level infections or weakly positive samples may remain undetected, impacting diagnostic accuracy and reliability [25].

#### 2.1.2. Multiplex PCR

**Figure 2:**
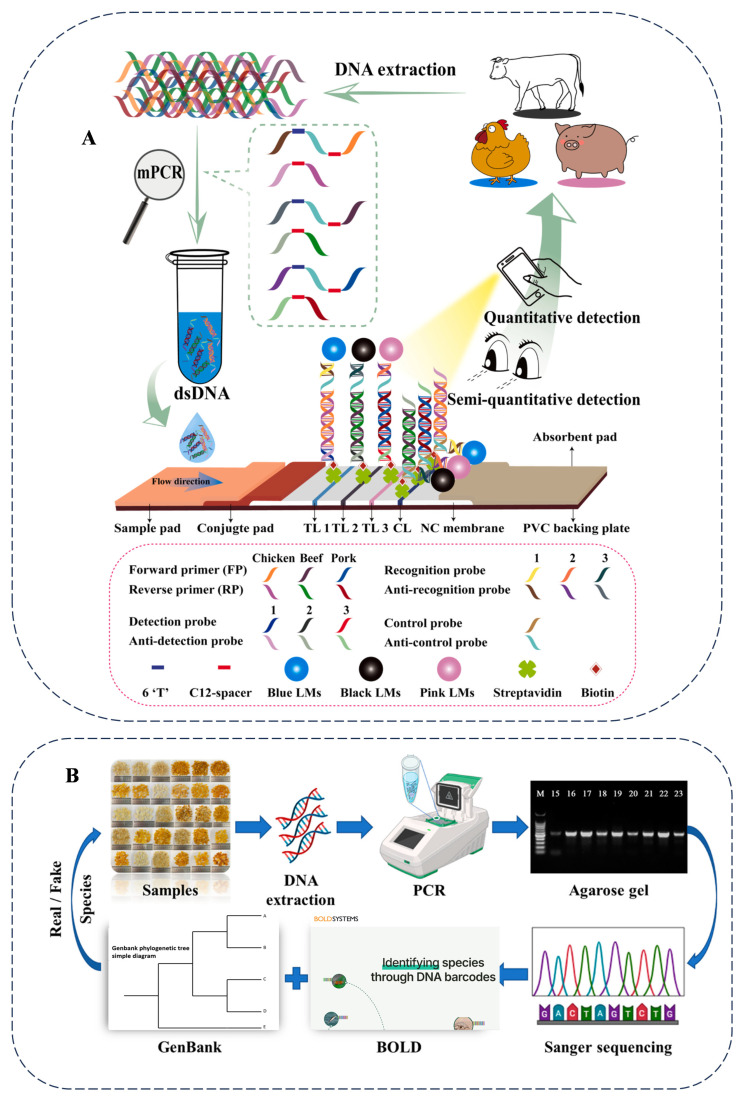
A schematic illustration of the mPCR-NALFS strategy for detecting beef, pork, and chicken (**A**) and DNA barcoding assay for the authentication of commercially available snow lotus seed products (**B**). Cited from references [28,53] with permission.

Multiplex PCR is another technology based on conventional PCR that can amplify multiple DNA fragments simultaneously by incorporating multiple pairs of primers into the same reaction system. This technology is primarily used to detect pathogenic microorganisms in food and identify food adulteration. Compared with other detection methods, the advantage of this protocol is that it can determine the harm to human and animal health and the zoonotic risk of samples in a single PCR. After combining the pre-enrichment step, its sensitivity is similar to that of real-time PCR, but it does not require expensive equipment and materials. However, this method involves multiple steps such as pre-enrichment and PCR, so the process is relatively complex, and the detection time is relatively long (6–8 h for pre-enrichment plus PCR time). It has high requirements for experimental operations, which may affect its detection efficiency and widespread application [26,27]. Multiplex PCR is particularly effective for detecting adulteration in meat, as it can accurately identify multiple types of meat, seafood, and processed products simultaneously. Meat from different animals was tested, as shown in Figure 2A. Although multiplex PCR is a rapid and accurate method for identifying food adulteration, it requires high-purity sample DNA, and interactions among species can lead to interference in the PCR [28].

#### 2.1.3. qRT-PCR

Real-time fluorescent quantitative PCR (qRT-PCR) is a method for the quantitative analysis of DNA. By incorporating fluorescent groups into the PCR system, the entire PCR process is monitored in real time through the accumulation of fluorescent signals [29]. Compared to conventional PCR, qRT-PCR enhances detection efficiency, sensitivity, and specificity, making it suitable for quantitative detection. In a study of gene expression in lemon balm, qRT-PCR had high sensitivity. It could accurately detect the subtle changes in the expression of the PAL gene after treatment with jasmonic acid and gibberellin. The results were accurate, allowing for the precise quantification of gene expression levels, and the method could meet the requirements of high-throughput research. However, it relied on reference genes. Different algorithms recommend different optimal reference gene combinations. Improper selection can lead to result deviations. The experimental cost is high, requiring specialized instruments and special reagents. Data analysis is also complex, demanding a high level of proficiency from experimental personnel.

Conventional PCR is simple to operate and easy to master. It has a relatively low cost, only requiring basic reagents and equipment, and is widely used, such as for gene amplification. However, it cannot accurately quantify gene expression and can only roughly estimate gene expression status. Its sensitivity is relatively low, which may result in the omission of information from low-expressing genes. Moreover, it is prone to contamination, and the products may interfere with the judgment of subsequent experimental results [30]. Currently, this technology has been implemented in food adulteration detection and has established corresponding detection standards. Real-time PCR has a wide range of applications, such as the identification of adulteration in meat products, seafood, and dairy products [31,32].

#### 2.1.4. ddPCR

ddPCR has rapidly advanced in recent years, offering greater sensitivity and accuracy compared to qRT-PCR and allowing for direct absolute quantitative analysis without the need for standard products. ddPCR is particularly suitable for studying small relative expression differences, low-abundance genes, and allele differential expression. For instance, ddPCR can detect soybean allergens in various food matrices, including meat products, flour, milk, and fatty creams [33,34]. Therefore, it can be used to distinguish the compositional differences in different species and thereby distinguish between species. In an experiment detecting porcine-derived components, the recombinant pUC57 plasmid carrying porcine-specific gene fragments was used as the amplification target. After gradient dilution, it was detected by qPCR and ddPCR. Both methods had high precision in absolute quantification and linearity evaluation, with R^2^ values of 0.9971 and 0.9998, respectively. However, their detection limits were different. The detection limit of ddPCR was one copy number per reaction, while that of qPCR was five copy numbers per reaction. ddPCR was more sensitive at low concentrations, and qPCR could detect porcine-derived DNA in various samples. qPCR is widely used and has acceptable precision. It can be carried out independently, but it relies on Cq analysis. Its precision is questionable at low concentrations and in complex matrices, and it also requires reference materials and standard curves. ddPCR can perform absolute quantification, has high precision and sensitivity, and is less affected by inhibitors. However, the costs of materials and instruments are high [35].

### 2.2. Next-Generation Sequencing (NGS)

NGS, also known as high-throughput sequencing, has been widely utilized in genomics research. Compared to traditional Sanger sequencing, NGS can determine hundreds of thousands to millions of DNA sequences, significantly improving sequencing efficiency [36]. In the field of microbial research, multiple next-generation sequencing (NGS) technologies provide diverse tools for the in-depth analysis of microbial communities. For instance, 16S rRNA gene amplicon sequencing targets the conserved regions of bacteria and archaea [37]. To conduct this method, collect samples of fermented foods, record relevant information, and perform pre-processing to isolate microbial cells. Extract the genomic DNA of microorganisms and assess the concentration and quality of the DNA. Specifically amplify the V4 hypervariable region of the 16S rRNA gene via PCR, and perform paired-end sequencing in the library using the Illumina high-throughput sequencing platform to generate a large amount of sequence data. Finally, conduct bioinformatics analysis to reveal the composition and diversity of the microbial community within the samples. This approach is cost-effective and easy to operate, making it suitable for rapidly assessing microbial diversity and community structure. However, its resolution is limited, and it is susceptible to primer bias.

Whole-genome sequencing (WGS) covers the entire genome without bias and can accurately identify microorganisms at the strain level. It plays a crucial role in pathogen tracing, drug-resistance research, and food contamination tracking [38]. Nevertheless, its high cost and complex data analysis processes limit its large-scale applications. Gene panel sequencing enriches specific gene regions through customized probes, striking a balance between cost and sequencing depth. It is suitable for studying known functional genes but relies on pre-existing sequence information and has limited flexibility. Shotgun metagenomics can directly perform random sequencing on the DNA of all microorganisms in a sample without PCR amplification, comprehensively revealing the composition and functions of microbial communities. It is particularly suitable for the discovery of unknown microorganisms [39]. However, its high cost and large data volume pose higher requirements for computing resources. Each of these technologies has its own advantages and disadvantages. Researchers need to select appropriate methods based on specific research objectives and resource conditions.

The primary NGS techniques include pyrosequencing. The base sequence is determined based on monitoring the release of pyrophosphate, and the read length is relatively long (www.roche.com). (accessed on 21 March 2025) Sequencing by synthesis by using fluorescently labeled nucleotides has the characteristics of high throughput and low cost (www.illumina.com). (accessed on 3 November 2024) Semiconductor sequencing detects hydrogen ion concentration changes using a semiconductor chip and is characterized by high speed and low cost (www.lifetechnologies.com). (accessed on 3 November 2024) Nanoball sequencing uses DNA nanoballs and combinatorial probe–anchor synthesis technology, featuring high throughput and long read lengths (www.genomics.cn). (accessed on 3 November 2024) Sequencing by ligation determines a sequence by relying on the ligase reaction and is mostly used in specific fields (www.appliedbiosystems.com). (accessed on 3 November 2024) These technologies differ from traditional NGS in terms of sequencing principles, throughput, read length, speed, cost, and application fields. Similarly to real-time quantitative PCR, NGS is suitable for diverse food identification and quantitative analysis [40]. NGS is capable of sequencing millions of DNA fragments simultaneously, achieving high-throughput sequencing, and thus far surpassing Sanger sequencing in terms of scalability [41]. Additionally, the sequencing cost of NGS has significantly decreased, making large-scale sequencing projects more economically feasible [42]. When it comes to handling complex mixtures, NGS can detect low-frequency variations through deep sequencing, giving it higher sensitivity and accuracy in dealing with complex mixtures. These advantages have led to the widespread application and development of NGS in genomics research and clinical applications.

As a popular spice, *Oregano* faces adulteration problems in the complex supply chain. NGS technology plays an important role in identifying *Oregano*. By extracting, preparing libraries, and sequencing the DNA of *Oregano* samples, and then comparing the sequences with a plant reference database containing 5432 species entries, the species nature of *Oregano* products and possible adulterated plant materials could be determined [43]. The study found that common adulterated plants in *Oregano* samples included *Sweet marjoram*, olive leaf, myrtle leaf, etc. Among the *Oregano* samples tested, the adulteration proportion of olive leaf as an exotic plant material was 27%, that of *Sweet marjoram* was 4%, and that of myrtle leaf was 1%. Although NGS can detect types of plant materials, it cannot detect endogenous non-aromatic substances or exogenous substances without DNA, and the DNA read percentage cannot be directly equivalent to the plant weight percentage. Therefore, it needs to be combined with orthogonal analysis methods such as nuclear magnetic resonance (NMR). First, use NGS to screen and identify the analyzed materials to calibrate the NMR database. Then, conduct an initial fingerprint analysis of *Oregano* samples through NMR to identify the type of *Oregano*, its geographical origin, and the addition of other plants. The combination of the two can more comprehensively and accurately identify the authenticity and adulteration of *Oregano*. Similarly, NGS was employed to identify species in highly processed and complex meat products. The findings indicated that the method accurately identified all animal components in pooled samples with detection limits as low as 1%, revealing six species with label discrepancies among 23 commercial animal products [44].

A method combining DNA metabarcoding with next-generation sequencing (NGS) was proposed to identify the plant and insect sources of honey. The method analyzes three specific gene regions: internal transcribed spacer 2 (ITS2) for pollen identification, ribulose-1,5-bisphosphate carboxylase/oxygenase large subunit (rbcL) for detecting trace plant DNA in non-pollen materials, and cytochrome c oxidase subunit I (COI) for bee species identification. Sequencing is performed using the Illumina MiSeq platform, and data are processed and analyzed with tools like QIIME and RDP Classifier to ensure sufficient depth and coverage. This approach successfully identifies honey sources in most cases but may face challenges with honey rich in polyphenols or crystallized honey, suggesting potential limitations in species identification accuracy under certain conditions [45].

Similarly, in a study, DNA was extracted from honey samples and the trnL-UAA gene region was amplified. The sequencing library was constructed and sequenced using the Ion Torrent platform. The data were filtered and analyzed with a bioinformatics pipeline containing over 150,000 reference sequences. The results identified plant DNA types and proportions in honey, revealing its plant-source composition. The trnL-UAA region was successfully amplified in all samples, generating 52,519 filtered reads. Read counts per sample ranged from 1467 (orange blossom honey) to 16,948 (linden honey), matching 254 plant species [46].

NGS technology shows significant advantages in detecting food adulteration. Its high-throughput capacity allows the simultaneous screening of DNA from multiple species, making it ideal for identifying multi-source ingredients in complex processed foods. It can detect trace adulteration as low as 0.1% through deep sequencing and analyze fragmented DNA using short-read technology. NGS can achieve precise species identification through whole-genome analysis, mitochondrial DNA, nuclear genes, or SNPs and even infer the geographical origin of raw materials using population genetics data. Unlike traditional PCR, NGS requires no pre-set primers for unbiased screening. Although the initial cost is high, its ability to process hundreds of samples in a single run makes it cost-effective for large-scale food testing [47]. NGS technology has remarkable advantages in food authenticity testing, especially in high-throughput and multi-target detection. Without prior knowledge of adulterated species, it can simultaneously analyze thousands of genomic regions. This makes it well suited for complex food matrices such as processed and mixed-ingredient foods to screen for potential adulterants.

There are various NGS platforms. Illumina, a widely used one, sequences DNA clusters using reversible fluorescent dideoxy terminators. Despite its short read lengths, it has high yields (e.g., HiSeq X Ten can generate up to 1.8 trillion bases), thus being suitable for large-scale, high-throughput detection (www.illumina.com) (accessed on 5 November 2024). PacBio’s single-molecule real-time sequencing offers long read lengths of up to 60,000 bases. Circularizing DNA molecules enhances the accuracy of complex genome analysis (www.pacb.com) (accessed on 5 November 2024). Oxford Nanopore’s nanopore technology, like the portable MinION, enables extremely long read lengths, yet error rates are still an issue (nanoporetech.com) (accessed on 5 November 2024). These different platforms provide flexible choices for food authenticity testing, satisfying diverse detection requirements.

Strong database support is an important foundation for the application of NGS technology [40]. Public databases such as GenBank, SRA (which stores over 8 petabytes of data), and RefSeq (covering curated sequences of over 84,000 species) and specialized databases like JRC GMO-Amplicons (containing over 240,000 genetically modified organism-related sequences) provide vast amounts of reference data for species identification. In addition, the construction of contemporaneous databases, which are targeted at specific commodities or geographical regions, can reduce the impact of environmental variables and further improve detection accuracy. NGS technology has shown high accuracy and sensitivity in meat species identification, effectively addressing the limitations of traditional methods in detecting multi-species mixtures or processed meats and providing a reliable approach for food authenticity verification.

### 2.3. DNA Barcoding

DNA barcoding is a species identification technology based on PCR and bioinformatics. It identifies unknown species in food samples by establishing specific mitochondrial DNA sequences and employing universal primers. In 2011, the U.S. Food and Drug Administration (FDA) approved DNA barcoding for species identification using standardized gene fragments to prevent seafood label fraud. For example, inspections revealed that some products labeled as “sturgeon caviar” were actually Mississippi pallid sturgeon (www.fda.gov) (accessed on 15 November 2024) Institutions like the Chinese Academy of Sciences are also promoting DNA barcoding for meat product identification, developing test kits and cloud computing platforms (www.cas.cn) (accessed on 15 November 2024) Certain regions of mitochondrial DNA contain relatively conserved sequences that allow for the design of universal primers, enabling efficient PCR amplification [48]. These regions also have sufficient variation to distinguish different species. Mitochondrial DNA primarily follows maternal inheritance and does not undergo recombination, making its genetic characteristics stable and reliable for species identification. Additionally, mitochondrial DNA has a relatively simple structure, with specific regions like the COI gene having an appropriate length for primer design, facilitating both PCR amplification and subsequent sequencing [49]. DNA barcoding is particularly effective in distinguishing mixtures of seafood from different species [50,51].

Moreover, DNA barcoding plays a significant role in high-value and value-added foods. To address the issue of ginseng product adulteration due to increased demand, a combination of DNA barcoding using rbcL, maturase K (matK), and ITS2 sequences, along with multiplex PCR, was employed [52]. The study examined 50 commercial ginseng supplements, revealing a success rate of 68% for DNA barcoding alone, 60% for multiplex PCR, and 72% for the combined approach. In the food industry, despite the high nutritional and market value of snow lotus seeds (SLSs), identification methods are lacking. As a high-value food, SLSs are often adulterated with the endosperm of other plant seeds due to the lack of effective detection standards and methods. A research team analyzed the homology of 11 ITS genes from six common Gleditsia plants, designed and screened universal primers, and constructed a DNA barcoding method using Sanger sequencing technology. Meanwhile, they optimized the sample pretreatment to extract the DNA of SLSs, which are rich in plant polysaccharides. The detection of 30 commercially available SLS products revealed that over 70% of the products were adulterated to varying degrees. Nearly two-thirds of the adulteration originated from plants of the same genus, 6.7% originated from Caesalpinia plants, and 30% of the products had added sucrose to increase the weight. By analyzing the homology of the 11 ITS genes among the six common Gleditsia species, universal primers suitable for these species were designed and screened. The DNA barcoding method constructed using these primers and the ITS genes could effectively distinguish the species origin of the SLSs. The research used this method to test commercial SLS products, successfully identified the species origin of the products, and uncovered new instances of food fraud. The DNA barcoding method established in this study can effectively identify the species of SLSs, providing technical support for combating food fraud, ensuring food safety, and promoting the healthy development of the SLS industry. Studies have demonstrated that the developed DNA barcoding method is effective for species identification in SLS samples (Figure 2B). This method is significant in combating SLS-related food fraud, ensuring food safety, and promoting the sustainable development of the SLS industry [53]. One study employed DNA barcoding technology, targeting the ITS region, a universal marker for fungi, to identify the species of nine commercially available dehydrated porcini (*Boletus* sp.) products [54]. By analyzing the ITS sequences of 108 samples and constructing phylogenetic trees, it was found that only 3 products contained European porcini (*B. edulis*). The remaining samples included *B. reticulatus* (the most common), *B. aereus*,* B. pinophilus*, and two Asian porcini species (*B. bainiugan* and *B. meiweiniuganjun*). Moreover, non-porcini species (such as *Imleria badia*) and the pathogenic fungus *Hypomyces chlorinigenus* were also present. The study confirmed that ITS barcoding could effectively expose product mislabeling and species admixture issues.

Thus, it can be concluded from the above that different types of food commonly use different key genes in barcode technology. For animal-derived food, the mitochondrial COI gene fragment is mainly used as the barcode. This gene fragment is about 650 bp long and can be effectively used for the identification of animal-derived food species. Plant-based food usually uses the chloroplast gene fragments rbcL and matK as DNA barcodes. For microbial food, such as fungi, the first choice for DNA barcoding is the internal transcribed spacer (ITS) of ribosomal DNA.

### 2.4. HRM

HRM analysis is an innovative technique for detecting gene mutations, genotyping, and SNPs. Its core principle is to utilize the melting characteristics of DNA sequences. The thermal stability of double-stranded DNA is affected by its length and base composition, and any change in the sequence will alter its melting behavior. HRM technology uses specific fluorescent dyes embedded into double-stranded DNA. As the temperature rises, the DNA gradually unwinds, the dye is released, and the fluorescence intensity decreases. By monitoring the changes in fluorescence intensity in real time during the heating process, a melting curve is generated. DNA fragments with different sequences produce different melting curve shapes due to their different melting temperatures (Tm) and the changes in fluorescence signals during the unwinding process, thus enabling the differentiation of genotypes. Due to the varying fragment lengths, GC contents, and GC distributions of different nucleic acid molecules, unique melting curves are generated during thermal denaturation, allowing for species identification through curve analysis [55]. One study employed HRM technology for species identification, collecting samples from wild and cultured *Mercenaria mercenaria*, *Mercenaria campechiensis*, and their hybrids. Following this, PCR amplification and HRM analysis produced derivative melting curves and estimated melting temperature points. The results from wild samples were validated through sequencing, and linear discriminant analysis and cross-validation were additionally performed. Ultimately, the prediction classification accuracy for each sample reached 100%, with the average classification success rate in cross-validation also being 100%. Based on the melting curve analysis of the 16S rRNA gene fragment, there were cases where the thermal denaturation characteristics (T_m_ values) of individual samples fell between the typical values of two clam species due to intraspecific SNPs, making it difficult to accurately group them. Intraspecific genetic variations could interfere with species discrimination based on thermal denaturation characteristics, as different species may exhibit similar thermal denaturation characteristics in certain gene fragments. The selection of fragments was mainly based on sequence differences, stability, and the research purpose and applicability. The target fragments needed to have significant sequence differences among different species. For example, there were multiple single-nucleotide polymorphisms in the cytochrome c oxidase subunit (CO1), 16S rRNA, and ITS1 gene fragments of *Mercenaria mercenaria* and *Mercenaria campechiensis*, which resulted in different GC/AT ratios, leading to obvious differences in melting curves and T_m_ values, and they could thus be used for species identification. In terms of stability, the target fragments had to be relatively stable to reduce the interference of intraspecific variations. Although the fragments selected in the research were generally effective, intraspecific SNPs still affected the accuracy. Therefore, in practice, multiple fragments are often combined to improve the accuracy of identification. Consequently, HRM is a low-cost, high-throughput, and rapid method that is not constrained by location, making it suitable for identifying various types of food adulteration. Furthermore, HRM technology has been utilized to verify wine authenticity. In the context of wine research, HRM plays a crucial role in addressing fraud within the wine industry and ensuring the authenticity and quality of wines, particularly in grape variety identification and wine analysis [56]. To facilitate variety identification, an HRM analysis method was developed for short-fragment SNP markers. Through HRM analysis of samples from diverse grape varieties, the study demonstrated that specific HRM-SNP assays could effectively distinguish between leaf and must DNA samples. Additionally, research on grape varieties such as Barbera, Dolcetto, and Arneis from northwestern Italy employed TaqMan and HRM methodologies for genotyping [57].

HRM is a flexible post-PCR method that can be used to identify SNPs, simple sequence repeats (SSRs), and other mutations, such as insertions and deletions (INDELs). It performs well in the same DNA region with multiple SNPs or INDELs, while TaqMan may not be able to detect effectively in such cases. SSR markers have inaccurate PCR amplification issues in wine detection because DNA degrades during fermentation and there are inhibitors such as polyphenols. SNPs, as markers, have higher stability and accuracy because they are biallelic with a low mutation rate and are suitable for low-quality DNA. From processing plant materials to SNP identification and subsequent experimental vinification, DNA extraction, quantification, and genotyping analysis indicates that TaqMan is more efficient for detecting single SNPs and excels in genotyping experimental and commercial wines. HRM analysis is a powerful tool for differentiating fungal and bacterial species and strains. In fungi, it uses markers like 18S rRNA, ITS, 26S rRNA, TEF1alpha, and yeast-specific sequences (e.g., circadian clock-associated 1, RNA polymerase II subunit B2) to distinguish strains. For example, the HRM analysis of 18S rRNA can differentiate Saccharomyces cerevisiae var. boulardii from S. cerevisiae, while RNA polymerase II subunit B2 analysis can distinguish probiotic strains from other S. cerevisiae strains. In bacteria, HRM analysis uses markers such as 16S rRNA, DNA gyrase subunit B, and RNA polymerase beta subunit to differentiate species like Escherichia coli and Salmonella. These examples highlight HRM analysis’s efficiency and broad applicability in microbial identification [58].

However, the HRM method has limitations in processing amplicons with single SNP mutations, which highlights the distinct applications and scenarios for using both methods. Additionally, other studies have focused on HRM analysis using SSR markers [59]. By collecting samples of specific grape varieties and extracting DNA, 12 SSR markers were analyzed, revealing that some new markers surpassed traditional markers in amplifying must and wine DNA. Subsequently, HRM analyses were conducted, demonstrating effectiveness in distinguishing must DNA samples. Although challenges persisted with varietal issues in wine DNA samples, HRM and other markers exhibited promising potential, thereby providing additional methodological options for grape variety identification.

### 2.5. Loop-Mediated Isothermal Amplification (LAMP)

Unlike PCR, LAMP is a widely utilized isothermal amplification reaction in scientific research. Compared to PCR, LAMP demonstrates higher sensitivity and specificity while exhibiting greater tolerance to PCR inhibitors. LAMP is a nucleic acid amplification technique that can be carried out at a constant temperature between 60 and 65 °C, eliminating the need for thermal cycling equipment. It uses four to six primers to target six to eight regions of the target gene, featuring high specificity and sensitivity. The reaction is catalyzed by strand-displacing DNA polymerase, such as bacillus stearothermophilus (Bst) DNA polymerase, resulting in the formation of stem–loop structures and multiple repeats of the target sequence. LAMP has advantages such as short amplification time, suitability for resource-limited settings, and applicability for point-of-care testing. The globalization of food supply chains has led to an increase in food fraud, creating an urgent need for rapid on-site detection systems. LAMP has extensive applications in the on-site detection of meat, plant, and dairy products [60].

Addressing the significant issue of meat adulteration and the limitations of existing detection methods, researchers developed a portable device that integrates LAMP with fluorescence and colorimetric dual-channel detection for the identification of pork adulteration in beef [61]. Specific primers were designed using porcine mitochondrial sequences obtained from GenBank. Sequence alignment was performed across multiple common pig breeds to ensure specificity. Sequence alignment was performed four times in multiple common pig breeds. Finally, the mitochondrial D-loop gene of *Sus scrofa* was selected as the detection target. The Primer V5 website (http://primerexplorer.jp/lampv5e/index.html) (accessed on 17 November 2024) was utilized to design LAMP-specific primers for the mitochondrial D-loop gene. After the design was completed, the researchers checked for potential cross-hybridization with the sequences of other species, and the LAMP reaction conditions were optimized. Hydroxynaphthol blue and calcein indicators were incorporated into the device to facilitate visual detection. The developed LAMP-PD device offered significant advantages in terms of cost, operation, and functionality. This device cost only USD 54.65, with detection limits for pork as low as 0.1% (colorimetric detection) and 0.5% (fluorescence detection). The detection results were comparable to those obtained using commercial instruments, and the tool offers significant advantages in resource-limited environments. This device integrates LAMP and colorimetric and fluorescence dual-channel detection functions. There is no need to transfer the reaction products to other containers for detection. Traditional PCR amplification requires specialized equipment such as thermal cyclers, while LAMP-PD integrates LAMP and detection functions. The device is relatively simple, reducing equipment costs and operational difficulties. Commercial instruments not only require complex transfer operations but also rely on professionals, making it difficult to meet the needs in resource-limited environments. Its colorimetric/fluorescence dual-channel detection mode can enhance the reliability of results, can determine the existence of specific DNA sequences, and has high detection sensitivity, which can meet the needs of meat adulteration detection. In resource-limited environments, such a multifunctional and sensitive detection device is of great significance.

To address the challenge of goat milk adulteration with cow milk, researchers constructed a microfluidic-integrated nucleic acid lateral flow strip (LFS) detection platform [62]. By modifying the loop primers of LAMP and substituting traditional expensive antibodies with the amplification terminator spacer C3 and oligonucleotide sequences, costs were reduced while multiple detections were enabled. Additionally, the integration of the LAMP and LFS detection steps into a microfluidic chip mitigated the risk of aerosol contamination. Testing showed that the platform demonstrated strong specificity for distinguishing goat and cow milk components, detecting as little as 1 pg of cow DNA and 5% cow milk adulteration in actual goat milk samples. Its detection limit was comparable to that of the qPCR method, with a cost of approximately USD 2 and a detection turnaround time of 50 min, making it suitable for the rapid on-site detection of dairy products.

Moreover, LAMP is effective in detecting meat, plant, and dairy products [63,64,65]. However, it has limitations like non-specific amplification, complex primer design, unsuitability for short sequences, and poor multiplex detection. To address these issues, engineered Bst DNA polymerases (e.g., Bst 2.0/3.0) and OmniAmp polymerases have been used to improve performance. Innovative primers (e.g., loop, stem, and SLP) enhance primer flexibility. Combining CRISPR–Cas cleavage, microfluidic chip detection, and two-stage recombinase polymerase amplification effectively solves problems like non-specific amplification and short-sequence detection, expanding their potential in pathogen detection and gene diagnosis [66].

### 2.6. Bubble-Mediated SEA (Strand Exchange Amplification)

**Figure 3:**
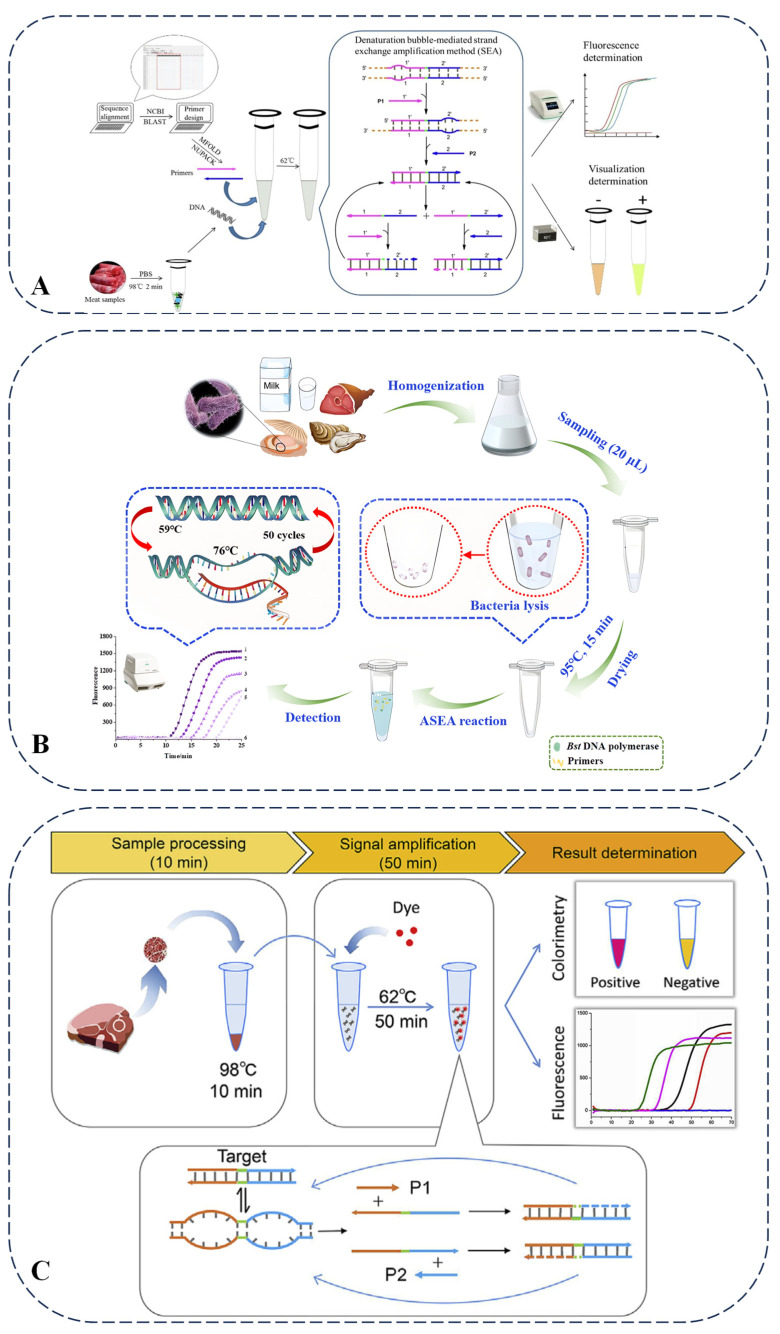
(**A**) Schematic illustration of SEA assay for meat sample adulteration [67]. (**B**) Scheme of pork detection by isothermal SEA method and (**C**) scheme of biphasic detection of S. enterica-contaminated food samples [70,71].

SEA is a form of isothermal nucleic acid amplification reaction. Unlike LAMP, SEA facilitates easier primer design and minimizes the likelihood of primer dimer formation. This technique operates at constant temperatures, negating the need for a thermal cycler, and further simplifies primer design, thereby reducing the occurrence of dimers. A direct and visual isothermal method (SEA) was developed for the detection of meat adulteration. Taking the detection of beef adulterated with duck meat as an example, this method targets the mitochondrial sequence of ducks. When detecting beef adulterated with duck meat, the Bst 2.0 WarmStart DNA polymerase used in this method belongs to the Bst enzyme family that relies on strand displacement. It is characterized by its ability to efficiently catalyze DNA amplification under isothermal conditions at 62 °C, which is highly suitable for the simple reaction system required by the SEA technique. The sample is lysed by heating at 98 °C for 2 min to release DNA, and then the reaction is carried out at 62 °C for 50 min. The results can be determined by fluorescence detection or by observing the color change with the naked eye. Its detection limit is as low as 10 pg/μL of duck DNA, and it can detect 0.1% adulteration in duck/beef mixtures. When combined with rapid DNA release technology, the entire detection time is shortened to 1 h, eliminating the need for a complex DNA extraction process. Compared with other nucleic acid-based meat adulteration detection methods, such as PCR, LAMP, and electrochemical sensors, the advantages of SEA lay in its high functional integration; it integrates sample processing, amplification, and visualization into a single process, eliminating the need for complex equipment or separate steps. It only requires a simple heating block and can be used for on-site detection. Its detection limit is 0.01 ng/L, with a small number of primers (only two), a short target fragment of 41 bp, and a suitable detection temperature, and the results can be read by the naked eye. However, PCR has higher sensitivity, with a detection limit of up to 0.01–0.08 ng/μL. Food adulteration detection usually requires high sensitivity to detect low levels of adulterated components. The PCR method can detect very low concentrations of adulterated components by amplifying target DNA sequences, so it has good application prospects in food adulteration detection. LAMP requires 4–6 primers, making the reaction system relatively complex (Figure 3A) [67]. The methods developed for detecting foodborne pathogens and DNA based on SEA technology present innovative detection principles. Direct detection in complex meat matrices may be delayed (e.g., with prolonged time to positive values) due to insufficient DNA release or matrix inhibition. Although it can detect 0.1% duck meat adulteration, its higher detection limit (10 pg/μL) compared to PCR and LAMP may restrict its use in extreme trace adulteration cases. When combined with the quantum dot bead-based immunochromatographic technique, SEA exhibits high sensitivity, good selectivity, and acceptable accuracy and precision [68]. When combined with the electrochemical aptamer sensor, the sensitivity is enhanced through nanomaterials, showing high specificity for SEA. It enables rapid detection, has a wide linear range, and is suitable for the detection of complex samples [69]. Furthermore, it demonstrates robust performance in terms of sensitivity, specificity, and detection time, as illustrated by Figure 3B,C, thereby providing substantial support for food safety measures [70,71].

### 2.7. CRISP–CAS System

As a cornerstone of modern biotechnology, gene editing technology has undergone significant development and evolution. From traditional genetic engineering methods such as transcription activator-like effector nucleases (TALENs) and zinc finger nucleases (ZFNs) to the now widely recognized CRISPR–Cas system, each technological advancement has led to breakthroughs in biological scientific research. With its distinct mechanism of action, CRISPR–Cas9/12 technology can precisely edit the genomes of organisms, heralding a new era of innovation in the food sector and establishing itself as a cutting-edge focus in recent research and applications.

**Figure 4:**
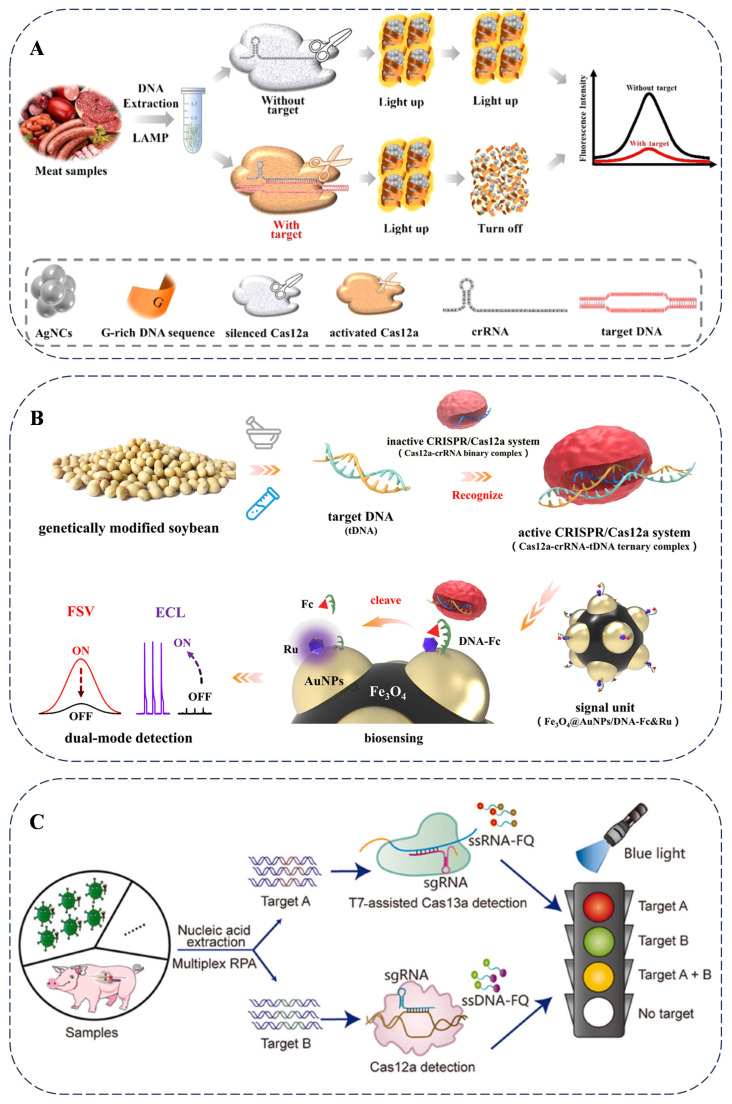
(**A**) Nucleic acid amplification-based methods for meat adulteration detection using LAMP reactions and DNA-templated silver nanoclusters using CRISPR-based assays [73]. (**B**) Nucleic acid amplification-free-based methods for genetically modified soybeans detection using CRISPR-based assays [74]. (**C**) A schematic of genome-edited pig detection with portable blue and ultraviolet light transilluminators [76].

As reported, CRISPR–Cas12a is utilized for authenticity detection in meat species. By integrating microdroplet technology and machine learning, it achieves the highly sensitive and specific detection of the pork-specific ND1 gene and the duck-specific IL-2 gene. This method can identify usually truncated DNA in highly processed meat products, demonstrating considerable application potential in food detection [72]. As shown, nucleic acid amplification-based methods (Figure 4A) and nucleic acid amplification-free-based methods (Figure 4B) are illustrated for food adulteration and genetically modified food product detection using CRISPR-based assays, which achieve a satisfactory, sensitive, and accurate determination [73,74]. Regarding the application of the CRISPR–Cas system in fungi and bacteria [75], in fungi, for example, in Saccharomyces cerevisiae, the CRISPR–Cas9 system is used to knock out the ADE2 gene, resulting in a red-colored phenotype. This allows for the screening of successfully edited strains. In bacteria, such as *Escherichia coli* and *Staphylococcus aureus*, the CRISPR–Cas9 system is employed to precisely knock out specific genes. In *E. coli*, the lacZ gene is targeted. Strains with successful editing are screened by detecting the activity of β-galactosidase. In *S. aureus*, the agr gene is the target, and strains with successful editing are screened by detecting the activity of plasma coagulase. Through these methods, different microorganisms can be distinguished.

Portable devices have been developed for the analysis of diverse types of food adulteration. Studies that utilize the Cas12a and Cas13a systems have established multiple detection strategies in combination with portable technologies. The rapid visualization CRISPR (RAVI–CRISPR) method enables rapid fluorescence imaging and allows for the visual identification of genome-edited pigs with 100% sensitivity and specificity (Figure 4C). The multi-CRISPR technique is designed to analyze co-infections and multiple gene edits in meat products simultaneously [76]. The advancement of on-site food authenticity identification methods provides real-time monitoring capabilities for the food supply chain, facilitating the early detection of food adulteration problems. The continuous development of portable detection technologies with multi-target recognition capabilities represents a promising direction for the analysis of various food products.

### 2.8. Other Techniques

In addition to the aforementioned methods, genomics research also encompasses biochip and biosensor technologies, third-generation sequencing (TGS), fourth-generation nanopore sequencing, and the integration of multiple genomic technologies along with other emerging techniques.

**Figure 5:**
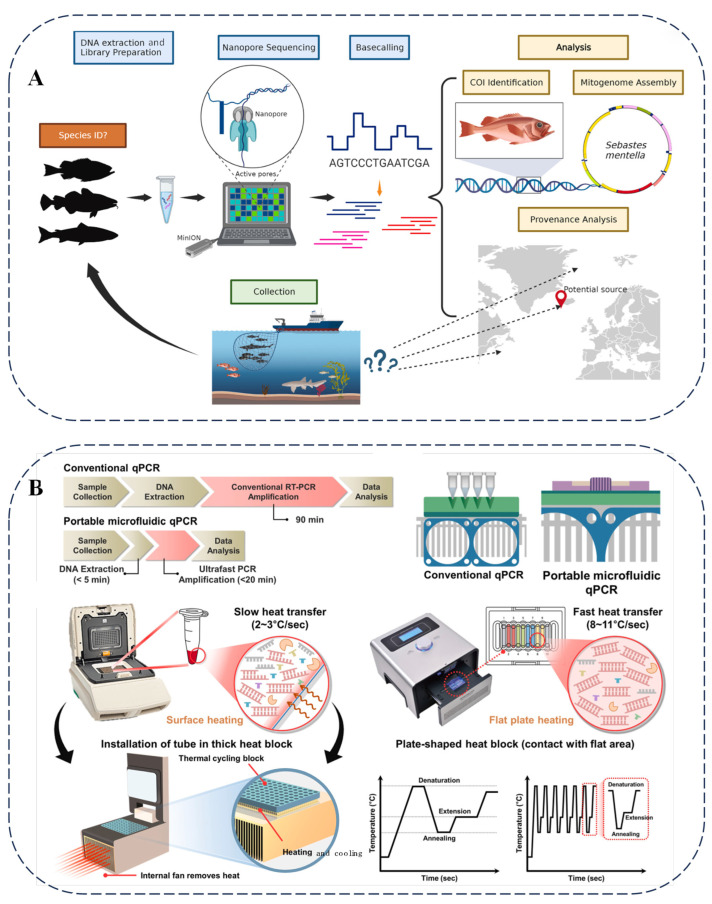
(**A**) Illustration of process of nanopore sequencing of seafood, specimens collected from unknown locations [77]. (**B**) Comprehensive concept and design of conventional and portable microfluidic qPCRs for detecting laver species [80].

Nanopore sequencing technology is playing an increasingly crucial role in food analysis. Researchers have utilized nanopore sequencing to examine seafood and marine wildlife [77]. By sequencing fish and shark samples from various regions, this technology has introduced innovations in monitoring seafood supply and wildlife trade (Figure 5A). Additionally, the metabarcoding capabilities of nanopore sequencing have been applied to detect commercial surimi products [78], revealing the presence of multiple fish species and highlighting instances of species substitution and adulteration in certain products.

In the field of food science, biosensor technology plays a crucial role. Glycan-coated magnetic nanoparticles combined with parallel plasmonic biosensors can efficiently detect *carbapenem-resistant Escherichia coli*, thus ensuring the safety of food and water [79]. Figure 5B presents the microfluidic chip qPCR method, which relies on specific primers and convenient equipment to effectively distinguish differences among seaweed species, thereby preventing food fraud and ensuring the quality of seaweed in the supply chain [80].

Emerging genomics research methods have made significant strides in the field of food science. From the innovative application of nanopore sequencing technology for food safety monitoring and species identification, as well as the important contributions of third- and fourth-generation sequencing technologies to gene function research and variety improvement [81], the development of these technologies has not only enhanced our understanding of the food system but also provided powerful tools for ensuring global food safety and advancing the food industry.

## 3. Identification of Food Product Authentication

Since differences in biotypes are primarily determined by variations in gene sequences, genomics-based food analysis methods have been widely adopted for food identification to combat adulteration. These approaches encompass the utilization of associated technologies, the selection of target genes, the application of molecular markers, and the identification of various food adulterants. The following sections will provide detailed descriptions of studies employing genomic techniques to assess food certification across diverse categories, including meat products, aquatic food products, dairy products, and oil products.

### 3.1. Meat Products

Meat products represent a substantial market share globally; however, the issue of meat adulteration is serious, as exemplified by the horsemeat scandal in Europe and prevalent adulteration of beef in China. Meat adulteration refers to the practice of substituting low-cost raw materials for original ingredients to enhance profit margins, such as adulterating beef with pork or mislabeling products deliberately [82,83].

Currently, the genes utilized for identifying meat adulteration primarily consist of mitochondrial sequences, including the cytochrome b (Cytb) gene, 12S rDNA, 16S rDNA, and the D-loop gene. Mitochondrial genes evolve more rapidly and are more abundant and stable compared to nuclear genes, making them suitable for distinguishing closely related species. Specific primers have been designed using the mitochondrial D-loop region, in combination with real-time PCR for detection. Meatball samples with varying proportions of monkey meat and beef mixtures were prepared. Through DNA extraction, primer design, real-time PCR, and sequencing analysis, the designed primers successfully identified monkey meat, achieving a limit of detection of 0.0078 ng (corresponding to 1% monkey meat in beef meatballs) with good repeatability. This approach offers an effective means for halal authentication [84].

**Figure 6:**
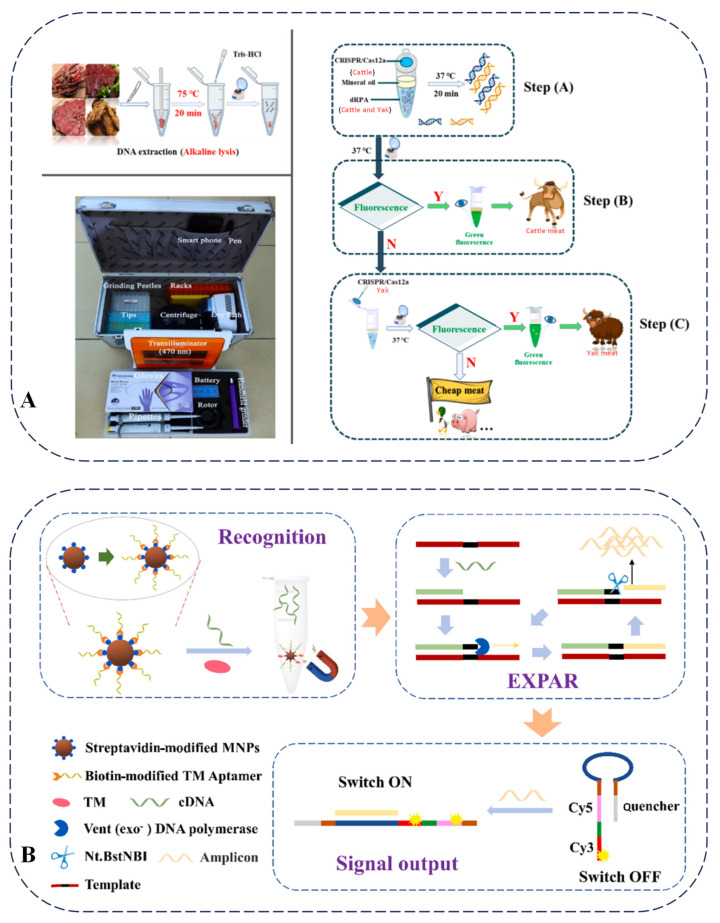
(**A**) Duplex recombinase polymerase amplification combined with CRISPR-Cas12a assay for on-site identification of yak meat adulteration [85]. (**B**) Schematic illustration of detection principle for shellfish TM by ratiometric fluorescent aptasensor based on EXPAR [89].

Given the high price of yak meat and the limitations of existing detection methods, a duplex recombinase polymerase amplification combined with a CRISPR–Cas12a strategy was established [85]. This method demonstrated high sensitivity for detecting yak and cattle DNA, effectively identifying low-content adulterated beef in both raw and cooked meat models, and achieved a detection limit of at least 1% (*w*/*w*) for yak meat under various adulterated conditions. Additionally, it was cost-effective and allowed for rapid on-site identification under mild reaction conditions (Figure 6A). The limitations of current genomic technologies in food authenticity detection mainly lay in their dependence on laboratory conditions, operation by professional personnel, and expensive equipment, especially in terms of rapid on-site detection. Future research directions should focus on developing more portable, rapid, and low-cost on-site detection technologies to meet the detection needs of different types of foods.

### 3.2. Aquatic Food Products

Fish and other seafood products are significant trading commodities in the market; however, due to limited public knowledge regarding various types of seafood, adulteration is a common issue. In addition to conventional Sanger sequencing in PCR, methods to identify adulteration in aquatic products predominantly include nanopore sequencing, molecular identification, and multiplex PCR [77,86].

A fast, reliable, and low-cost method for identifying fish species from the *Mullidae* family traded in the eastern Mediterranean was developed [87]. To address issues such as mislabeling and difficulties in the morphological identification of seafood products, the study was based on the SNPs of the mitochondrial cytochrome CO1 and CytB genes. Species-specific primers were designed to generate fragments of different lengths or produce specific melting curves in agarose gel electrophoresis. The detection limit evaluation showed that, except for *M. surmuletus*, which required at least 10 pg of template DNA for reliable results, the linear dynamic range of both singleplex and multiplex qPCR for other species was from 10 ng to 1 pg. These methods are highly specific and sensitive, can complete detection within 80 min, and are cost-effective. They are suitable for the rapid routine identification of large-scale samples and help combat fraud in the fish trade.

*Crustacean shellfish* are nutritious but can induce allergic reactions. A previous study highlighted the current status of *Crustacean shellfish* allergies and emphasized the necessity for the strict avoidance of consumption and accurate detection of allergens. Various detection methods, including those based on antibodies, mass spectrometry, and DNA, were introduced, with an analysis of their respective advantages and disadvantages [88]. For instance, while DNA methods offer significant benefits, they also have limitations in detecting processed foods. In antibody-based methods, the detection limit of enzyme-linked immunosorbent assay is usually 0.01–1 mg/kg, which can achieve quantitative detection. However, it is vulnerable to the impact of processing, resulting in false negatives. The detection limit of lateral flow assay is 0.01–10 mg/kg. It is characterized by rapidity and portability and is suitable for on-site screening. The detection limit of immunosensors can reach 28.16 fg/mL. Although they have high sensitivity, their anti-interference ability needs to be optimized. DNA-based methods, such as real-time PCR, have a detection limit as low as 0.1 pg DNA. They have good applicability to processed foods, but attention should be paid to species specificity and cross-reaction issues. The detection limit of mass spectrometry methods is 0.1–25 mg/kg. These methods can detect multiple allergens simultaneously, but the equipment is expensive and the operation is complex. Additionally, a ratiometric fluorescent aptasensor based on exponential amplification reaction (EXPAR) was constructed to detect tropomyosin (TM) in food. This sensor employed a TM-specific aptamer as the recognition probe. By leveraging the competitive binding between TM and complementary DNA to the aptamer-magnetic nanoparticles, EXPAR was triggered to produce amplicons, facilitating fluorescence resonance energy transfer between Cy5 and Cy3 (Figure 6B). This sensor demonstrated the effective detection of seven shellfish samples and could provide technical support for allergen labeling supervision [89]. The detection of allergens in complex samples is limited by sample pretreatment and data processing methods, and there are challenges in quantitative detection. Future research directions may include the development of more efficient sample pretreatment technologies, the optimization of data processing algorithms, and the combination with other detection technologies to improve the accuracy, sensitivity, and quantitative ability of detection.

### 3.3. Milk and Dairy Products

**Figure 7:**
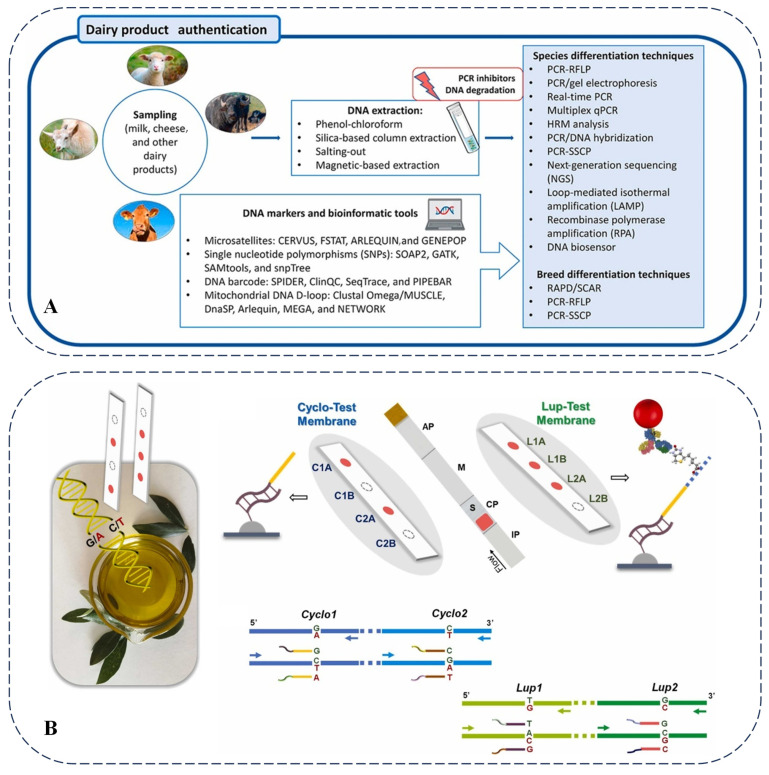
(**A**) Common molecular methods for detecting dairy products [91]. (**B**) Scheme of DNA analysis of olive oil and architecture and sensing mechanism of two devices used for detection by naked eye [95].

The adulteration of cheese with cheaper milk, false ingredients, and even harmful substances such as melamine constitutes one of the primary challenges in the dairy industry. In this context, various methods have been employed to detect the adulteration of dairy products. Recently, low-cost conventional PCR, high-specificity real-time PCR, and multiplex PCR have become the most common detection techniques for dairy products [90]. Figure 7A provides a detailed overview of several common molecular methods for identifying dairy products. The most commonly utilized molecular techniques include PCR, real-time PCR, multiplex PCR, and PCR-RFLP [91].

Moreover, relatively low-cost and quantitative methods are also employed for dairy product identification. The widespread issue of adulteration in the dairy industry, driven by economic interests, has created a demand for reliable detection methods. A comparison of the advantages and disadvantages of PCR-based technologies for authenticating dairy products—such as PCR-RFLP, multiplex PCR, real-time fluorescence quantitative PCR, digital PCR, and random amplified polymorphic DNA PCR—was conducted [24].

One study developed a double-tube multiplex TaqMan real-time PCR method that could simultaneously detect eight animal-derived ingredients, including cow, buffalo, goat, sheep, camel, yak, horse, and donkey products, in milk and dairy products [92]. This method innovatively used a pair of universal primers combined with two pairs of specific primers to construct a four-probe double-tube detection system. Verified by commercial samples, 38.75% of products with label discrepancies were successfully detected, effectively identifying the adulteration of unlabeled cheap milk sources such as cow and goat. This technology breaks through the limitations of traditional methods and has the advantages of high throughput, high specificity, and low cost, providing an efficient technical means for authenticity identification in dairy products. When detecting dairy product adulteration, the sensitivity and specificity of detecting target DNA in complex mixtures may be insufficient, and the cost and time efficiency still need to be improved when dealing with a large number of samples. Future research directions could focus on developing more efficient, sensitive, and cost-effective detection methods, as well as optimizing existing technologies to reduce false positive and false negative results. Meanwhile, efforts should be made to enhance the ability to detect multiple target components simultaneously.

### 3.4. Oils Products

Edible oil is an essential consumer good for humanity. Currently, the adulteration of edible oil is a pervasive global issue. This includes practices such as mixing inferior oils with high-quality oils to maximize profits, the use of unlabeled genetically modified oils, and the continued use of recycled oils. PCR is a molecular biology technique employed to detect the adulteration of edible oils and fats. As reported, various PCR methods can identify adulterated species by amplifying specific DNA sequences and analyzing nuclear and mitochondrial DNA. For instance, the RFLP analysis of the mitochondrial Cytb gene can be utilized to identify pig fat samples [93].

To reduce costs, olive oil is frequently mixed with other oils. As olive oil is often adulterated with inexpensive vegetable oils and existing detection methods exhibit limitations, a DNA sensor capable of simultaneously identifying seven plant species (including olives) was developed [94]. Following DNA extraction, PCR amplification, and plant discrimination reactions, functionalized gold nanoparticles form red spots on the sensor to indicate the presence of particular plant species. This method can detect proportions of adulterants as low as 5–10% with high specificity and reproducibility, offering the advantages of simplicity, rapidity, cost-effectiveness, and portability, thereby addressing both the detection of olive oil adulteration and the identification of the adulterating vegetable oils. Additionally, identifying olive oil varieties is critical for ensuring product quality and preventing fraud. A new DNA sensing device was developed that uses multiplex PCR to utilize specific primers for amplifying alleles at gene loci related to olive varieties (Figure 7B). This method demonstrates accurate and reproducible detection of olive leaves and oil samples and is expected to be widely applicable in the traceability of the plant origins of agricultural products and pharmaceuticals [95]. Currently, there are some limitations in the application of genomic technologies in olive oil detection. For example, the DNA extraction and purification steps may affect the accuracy and sensitivity of the detection. Additionally, there are some cross-reactions in multi-species DNA sensors, resulting in less-than-ideal specificity and sensitivity. Future research directions could focus on developing simpler and more efficient DNA extraction and purification methods, as well as further optimizing primer design and reaction conditions to improve the specificity and sensitivity of the detection. At the same time, it is also possible to explore the combination of genomic technologies with other detection technologies to achieve more comprehensive and accurate detection of olive oil.

### 3.5. Other Food Products

To safeguard the health of individuals allergic to *pistachios*, a single-tube nested real-time PCR method was developed [96]. This method is capable of detecting 1 pg of *Pistachio* DNA and can identify *pistachio* at specific concentrations within food matrices, such as wheat and ice cream. Its high sensitivity provides an effective means for detecting *pistachio* food allergens. Addressing the issue of adulteration in the *Pleurotus eryngii* market, s9ap was identified as an endogenous reference gene for detecting *Pleurotus eryngii* [97]. Qualitative and quantitative PCR analyses established a detection limit of 400 pg, offering essential genetic support for the development of a detection system for *Pleurotus eryngii*. Given the toxicity of *Gelsemium elegans* and its frequent adulteration in food and honey, a real-time PCR method combined with matK barcode technology was employed for detection. This method could detect as little as 0.1% *Gelsemium elegans* components, providing a reliable detection strategy to ensure food safety [98].

Due to potential confusion in distinguishing *Bacillus licheniformis* from *Bacillus paralicheniformis* within the Bacillus genus—and considering the latter’s ability to produce the antibacterial compound bacitracin, which poses a risk to food safety—a study analyzed data from 27 technical dossiers. The analysis revealed that 15 strains were taxonomically classified using 16S rRNA gene analysis, while 12 strains underwent WGS [99]. Ultimately, only the 12 strains analyzed by WGS could be unequivocally identified as *Bacillus licheniformis*, emphasizing the critical importance of accurate identification in evaluating food enzyme-producing strains to ensure food safety. To explore strains with potential probiotic properties in various foods, 646 bacterial strains were isolated and initially identified through 16S rRNA sequencing analysis. These strains were screened according to safety tests recommended by the FAO/WHO, culminating in the selection of eight strains (including *Lactiplantibacillus plantarum*, *Lacticaseibacillus paracasei*, and *Enterococcus faecium*) that exhibited diverse characteristics, with some demonstrating notable probiotic potential [100].

Recognizing the potential of insects as future food sources and the necessity for food authenticity testing, a DNA metabarcoding method was developed. Primers targeting the mitochondrial 16S rDNA fragment were designed using the Illumina platform, and various insect samples and food products were tested. The results indicated that this method exhibited high sensitivity in pure insect samples, processed model foods (such as insect cookies and burgers), and commercially available food products containing insect ingredients. The detection limit could reach 0.1% (*w*/*w*), and it could still accurately identify four edible insect species approved by the EU, such as the yellow mealworm (*Tenebrio molitor*) and the house cricket *(Acheta domesticus*), even in complex matrices. The study verified the applicability of this method in food authenticity testing, especially for screening trace insect components in highly processed foods, providing a reliable technical means to ensure food quality and safety [101].

## 4. Challenges and Prospectives

Genomics technology holds significant promise for the field of food identification. By analyzing the DNA sequences of organisms present in food, genomics technology plays an indispensable role in several key areas, including species identification, origin tracing, and adulteration detection. Its detection accuracy and sensitivity are expected to improve continuously. The optimization of NGS technology will facilitate the detection of even subtle changes in genetic materials present in food. Low-content genetically modified ingredients, trace levels of pathogenic microbial contamination, and minor instances of species adulteration will all be detectable [102,103].

Furthermore, genomics will be deeply integrated with multi-omics technologies, such as proteomics and metabolomics. Through comprehensive analyses from multiple perspectives—including gene expression, protein alterations, and metabolite profiles—it will provide a more robust and reliable foundation for food identification and quality assessment [104]. Advances will trend towards high-throughput and rapid detection methods, allowing for the testing of large quantities of food samples in a short timeframe to satisfy the demands of rapid screening and real-time monitoring in food production and distribution. Genomics can not only identify common food species but also accurately distinguish rare and easily confused species as well as new food resources [105]. Moreover, it can assess quality and potential contamination risks through the genomic analysis of microbial communities within production environments. Based on individual genetic characteristics, personalized food customization, nutritional assessment services, and specialized medical diet, programs can be offered to consumers [14,106]. In the integration of genomics-based food surveys using portable devices in water quality monitoring, researchers conducted metagenomic analysis of water samples from the UK and Ethiopia using devices such as mini-vacuum pumps [107]. The sequencing could be completed within one day, and the data could be obtained within 24–72 h. This analysis could distinguish different water samples and identify pollution indicators and hazards, and the results were verified by quantitative PCR. In cassava research, portable visible/near-infrared spectroscopy (Vis/NIRS) was combined with the RF model to predict the carotenoid content [108]. A total of 594 clones are evaluated, genetic correlations were studied, GWAS and genomic predictions were carried out, relevant genomic regions and genes were identified, and the accuracy of different models was compared, demonstrating the value of this technology.

Despite its potential, genomics technology encounters significant challenges in its application to food identification. Urgent improvements are needed in the standardization and regulation of the technology. Currently, issues such as inconsistent standards and non-standardized operational procedures persist. Establishing a comprehensive standard and quality control system is essential. In the face of massive and complex data, the development and optimization of bioinformatics algorithms and tools are necessary for effective analysis, interpretation, and the extraction of insights [109,110]. There are some examples and gaps in the application of genomic technology within the existing regulatory framework. For instance, the agricultural field of the United States is jointly regulated by three departments: the United States Department of Agriculture (USDA), the Environmental Protection Agency (EPA), and the FDA. In the medical field, when gene editing technology is used for treatment, it is also subject to strict regulation. However, there are issues such as a lag in technological updates within the regulatory framework, a lack of coordination in international regulation, and insufficient public participation and communication. The development speed of new gene editing technologies has outpaced the update speed of the regulatory framework, posing challenges for regulatory authorities when formulating safety evaluation plans and regulatory strategies [111].

Cost also presents a major barrier. The high expenses associated with certain technologies and equipment limit their widespread application. Therefore, it is crucial to reduce costs, develop economical and user-friendly detection methods and equipment, and enhance promotion and training efforts. In genomic surveillance, high costs are mainly due to equipment procurement and maintenance, reagents, local computing resources, and personnel training. Scaling up sample throughput (e.g., from 600 to 5000) can significantly reduce unit costs (66% for Illumina, 78% for ONT). Optimizing instrument utilization (e.g., increasing loading rates from 28% to 99%) reduces resource waste. Cost-saving strategies include choosing adaptable platforms (e.g., Illumina MiniSeq for low-throughput labs), sequencing multiple pathogens, using elastic cloud computing, sharing equipment in regional centers, and bulk purchasing. Decentralized sequencing networks and distributed data storage optimize resource allocation. Combining open-source tools with third-party data analysis outsourcing relieves bioinformatics pressure, achieving sustainable cost optimization [112].

Furthermore, ethical and legal considerations must be addressed, including the protection of personal genetic information privacy and the regulation of genetically edited foods. Improving relevant laws, regulations, and ethical guidelines is essential to standardize the application and management of this technology [112,113]. The issue of genomic data privacy is becoming increasingly prominent in personalized food recommendations and the certification of origin statements. With the popularization of genomic sequencing technology and the reduction in costs, a large amount of genomic data are being generated and stored, and the protection of their privacy has become a key challenge. In the field of personalized food recommendations, the use of genomic data can provide precise nutritional advice. However, data breaches may expose individual genetic information, triggering health and ethical issues. In the certification of food origin statements, genomic data can trace the source of food to ensure its quality and authenticity [114]. Nevertheless, improper use may lead to the leakage of the privacy of enterprises or regions, affecting their market competitiveness [115]. Therefore, ensuring the privacy of genomic data is of great significance for safeguarding individual rights and interests and promoting the sustainable development of the food industry.

Genome-based food quality detection methods face many challenges in sample quantification, which mainly originate from various factors during sample processing. In the detection of microbial food safety, diagnostic metagenomics is expected to replace traditional molecular methods [116], but there are some problems in sample quantification. At present, sample preparation methods are usually optimized for quantitative PCR and may not be suitable for metagenomic sequencing. Different DNA extraction methods used in experiments will lead to different bacterial compositions and inhibitors being extracted. DNA extraction methods significantly influence the results of metagenomic diversity. This is because different methods have varying extraction efficiencies for bacteria and may introduce inhibitors or damage DNA. For example, QIAamp and Easy-DNA showed large differences in the DNA concentration of fecal samples and inconsistent detection results for *Campylobacter jejuni* and *Salmonella typhimurium*. In food safety testing, a 10-g sample extracted by QIAamp could allow the detection of low-concentration target bacteria by qPCR but not by sequencing, while a sample extracted by Easy-DNA was found to be negative by qPCR due to inhibition but positive by sequencing. Differences in sample pretreatment and volume also lead to inconsistent results, highlighting the crucial role of extraction methods in microbial diversity analysis and food safety testing. Moreover, the results of qPCR and metagenomic sequencing may not be consistent, which may be due to the inhibitory factors in both methods. Parameters such as DNA concentration, fragmentation degree, and sequencing data volume in a sample have no linear or hierarchical correlation with the detection ratio of target microorganisms in metagenomic sequencing, making it difficult to optimize the sample preparation and sequencing processes through conventional quality parameters.

Traditional breeding methods have issues such as long breeding cycles and reduced genetic diversity [117]. Their samples possess complex genetic diversity and are influenced by the complex interactions among the genotype, the environment, and management factors, making it difficult to fully realize the yield potential. Although certain progress has been made in genomic research, and the availability of high-quality reference genomes has accelerated the discovery of genes and quantitative trait loci as well as marker development, current genomic tools are still insufficient to fully understand the extensive genetic variations in crop germplasm resources. High-throughput phenotyping technologies also have limitations and cannot accurately estimate complex quantitative traits.

In the future, genomics technology is poised for deep integration with various cutting-edge technologies. By combining genomics with nanotechnology, it is anticipated that more sensitive and portable nanoscale biosensors will be developed, enabling the rapid and accurate detection of trace components in food. The incorporation of artificial intelligence, particularly machine learning algorithms, will facilitate the efficient analysis of complex genomic data and the recognition of patterns, significantly enhancing the accuracy and efficiency of food identification. Training deep neural network models can not only improve these metrics but also reveal subtle features and underlying patterns that traditional methods often overlook [118,119,120]. Additionally, deep learning models can integrate genomic and spectral data to quickly and accurately determine whether food is genetically modified, identify superior varieties, and verify the authenticity of origins, among other applications. This integrated approach provides a comprehensive and high-precision solution for food identification, thereby promoting the high-quality development of the food industry while safeguarding food safety and protecting consumer rights [121,122].

Spectral technology offers the capability to rapidly acquire characteristic physical and chemical information about food. For instance, near-infrared spectroscopy can reflect the composition of food. By integrating spectral analysis with genomic data and examining the correlation between genetic traits and spectral properties, a more robust food identification model can be established [123,124]. Furthermore, the integration of Internet of Things (IoT) technology can facilitate the establishment of a comprehensive food monitoring network. This network can collect real-time genomic information related to food from farmland to the table, enabling the full-process traceability of food quality and safety.

In terms of detection accuracy, advancements in technology enable the detection of exceedingly subtle genetic changes in food. This includes the identification of low levels of genetically modified ingredients, minimal pathogenic microbial contamination, and concealed species adulteration [125,126,127]. Nanosensors show great potential in the field of food safety, such as in the real-time detection of foodborne pathogens in perishable goods. Specifically, colorimetric sensors based on gold nanoparticles can rapidly identify target DNA sequences through color changes. For example, they can detect *Staphylococcus aureus* in milk with a detection limit as low as 2.5 ng/μL [128]. This rapid and low-cost detection method is particularly suitable for on-site testing in resource-limited settings. Looking ahead, nanosensors can be combined with miniaturization technology to develop portable devices for real-time monitoring from farms to tables. For instance, a network of nanosensors can be embedded in the cold chain logistics to detect pathogens in perishable foods in real time and transmit data to a monitoring platform via wireless communication technology, providing timely warnings of potential risks and effectively preventing the spread of contamination.

The combination of blockchain technology and genomic traceability offers innovative solutions for enhancing the transparency of the food supply chain. Take IBM Food Trust as an example; it records every link of food from production to consumption through blockchain to ensure the immutability of data [129]. When combined with genomic data, it can further verify information such as the variety and origin of food. For example, by using blockchain to record the genomic data of grapes, the authenticity of wine and its origin can be ensured. In the future, the combination of blockchain and genomic traceability can be further deepened. For example, smart contracts can be integrated to achieve automatic authentication and traceability, improving efficiency and reducing costs. At the same time, with the development of genomic sequencing technology, more genomic information about food will be incorporated into the blockchain system, providing consumers with more comprehensive food information and enhancing trust in the food supply chain.

Genomics technologies demonstrate great potential and application value in feed identification. The applications of DNA-based technologies in the detection of the authenticity and traceability of plant-derived foods, feeds, and pharmaceuticals emphasize how these technologies help prevent fraud and adulteration in agricultural and food products [130]. Emerging technologies like the CRISPR–Cas system further enhance the sensitivity and efficiency of detection, providing stronger support for quality and safety management in the feed industry and helping to maintain market order and consumer trust. By using real-time PCR technology to identify chicken and pork DNA in dog food, it was found that 65% of the tested dog foods contained undeclared chicken DNA, and 41% contained undeclared pork DNA, revealing the common problem of adulteration with animal proteins of lower economic value in hypoallergenic and single-protein dog foods [131]. This technology is of great significance for evaluating the effectiveness of elimination diets for dogs with food allergies and helps to ensure the safety of pet food and the accuracy of clinical diagnoses.

To address the demands for rapid screening and real-time monitoring in food production and distribution, genomics technology is expected to realize high-throughput rapid detection in the future, allowing for the analysis of a large number of food samples within a short period. This will significantly enhance detection efficiency. Additionally, based on individual genetic characteristics, genomics technology can offer consumers personalized food customization, nutritional assessments, and specialized dietary services tailored to different populations. In summary, genomics technology holds great potential for future developments in the food industry and plays a crucial role in enhancing food quality and traceability analysis.

## Figures and Tables

**Table 1:** Differences among traditional PCR technology and its extensions.

PCR Types	Time	Cost	Accuracy	Complexity	Applications	Ref.
**Conventional PCR**	Relatively fast, typically completed within a few hours	Relatively low, suitable for large-scale experiments	Lower, relies on gel electrophoresis analysis	Simple, relatively easy operation	Genotyping, cloning verification, etc.	[20]
**PCR-RFLP**	Slower due to additional enzymatic digestion steps	Moderate, requires purchasing restriction enzymes	Moderate, limited by presence of restriction sites	Moderate, involves additional enzymatic digestion steps	Genotyping, genetic disease detection, etc.	[21]
**qRT-PCR**	Faster with real-time monitoring	Higher, needs specialized equipment and reagents	High, precise quantification through fluorescence signals	Moderate to high, requires sophisticated instruments and data analysis	Gene expression analysis, viral load determination, etc.	[22]
**Multiplex PCR**	Can be time-consuming due to optimization of multiple primer pairs	Higher, especially when using multiple fluorescent probes	Moderate to high, depends on primer design and reaction conditions	High, complex primer design and optimization required	Pathogen detection, multi-gene expression analysis, etc.	[23]
**ddPCR**	Longer analysis process because of need to generate and analyze numerous droplets	High, requires special equipment and reagents	High, capable of absolute quantification	High, technically demanding	Absolute quantification, rare mutation detection, etc.	[21]

## Data Availability

No new data were created or analyzed in this study.
